# Oligometastatic neuroendocrine tumors: characteristics and prognosis

**DOI:** 10.1038/s41416-026-03516-9

**Published:** 2026-07-06

**Authors:** Philipp Melhorn, Julia Postmann, Markus Raderer, Barbara Kiesewetter

**Affiliations:** Division of Oncology, Department of Medicine I, Medical University of Vienna, Vienna, Austria

**Keywords:** Neuroendocrine cancer, Metastasis

## Abstract

**Introduction:**

Oligometastatic disease (OMD) in neuroendocrine tumors (NET) has hardly been investigated so far and it is unclear how OMD should influence treatment decisions.

**Methods:**

The primary objective of this retrospective analysis was to evaluate characteristics and prognosis of the OMD state in patients with NET G1/G2 of gastroenteropancreatic origin.

**Results:**

The overall study cohort comprised 190 patients. OMD occurred in 29% of patients (*n* = 55), 58% of those (*n* = 32) had synchronous oligometastases. Oligometastases were limited to one organ in most patients (95%) and primarily involved the liver (83% or *n* = 43/52). Median overall survival (OS) from diagnosis was 117.5 months (95% CI 102.4–149.1 months). Calculated from first metastasis, median OS was 102.4 months (95% CI 98.3–117.5 months) and not significantly longer in oligometastatic patients (117.5 months) versus polymetastatic patients (100.2 months). Locoregional treatment of oligometastases was performed in 24/55 patients (44%) and showed a non-significant trend towards superior survival compared to no such treatment (125.4 versus 99.7 months).

**Conclusions:**

These results suggest that a low absolute number of metastases alone may not be a clear prognostic factor in NET. It remains unknown if locoregional therapies can achieve long-term disease-free survival in a significant number of oligometastatic NET patients.

## Introduction

Oligometastatic disease (OMD) is not well characterized in neuroendocrine tumors (NET), yet current guidelines use this term in recommendations regarding locoregional treatment [1, 2]. The natural history of well-differentiated NET can be relatively long, with current treatment options leading to a 10-year overall survival (OS) rate of roughly 60% [3]. Distant metastases represent an important prognostic factor in NET, apart from variables such as stage, histologic grade (NET G1-G3) and primary site (e.g., gastrointestinal, pancreatic, or pulmonary NET) [4]. Liver involvement represents the most common presentation of distant metastases in NET, with 82% of metastatic patients exhibiting liver metastases (versus 59% of patients with adenocarcinoma) [5]. Relevant for treatment, about 10–30% of patients show clinical symptoms related to hormone excess (e.g., carcinoid syndrome or insulinoma symptoms) [6].

The OMD paradigm was first proposed by Hellman and Weichselbaum in 1995 and describes an intermediate stage of cancer progression with limited metastases amenable to curative-intent treatment [7]. The model can be extended by dynamic disease transitions such as oligorecurrence, oligoprogression, and oligopersistence, which might be suitable for local therapy, but otherwise it is generally accepted that systemic progression requires systemic treatment [8]. OMD definitions are heterogeneous and commonly include an upper limit of 3 or 5 oligometastases, which also depends on the technical feasibility of resection or safe applicability of radiation [9]. This paradigm was tested in a randomized phase 2 trial called SABR-COMET, demonstrating significantly longer OS with stereotactic ablative radiotherapy (SABR, median OS of 50 months) versus the control arm (standard palliative radiotherapy, 28 months) in a cohort of 99 patients with different oligometastatic cancers [10].

With regards to NET, current European guidelines recommend considering surgical resection or locoregional therapies such as radiofrequency ablation (RFA) or transarterial embolization (TAE) for hepatic metastases [11]. Given the lack of informative studies, the long-term survival benefit of liver resection is unclear (10-year OS rate of 35–79%, but only 20–57% have complete resection and up to 94% experience local recurrence at 5 years) [12]. Importantly, besides the chance of radiological remission, (debulking) surgery of liver metastases can also achieve palliation of functional symptoms [13]. Under certain circumstances, even liver transplantation has been performed for hepatic metastases in NET [14]. According to a 2015 retrospective study, neoadjuvant peptide receptor radionuclide therapy (PPRT) can also be considered in initially unresectable or oligometastatic patients [15]. SABR is also gaining traction in the field of NET, with one recent report of 21 patients describing the sequential combination of PPRT and SABR, achieving low rates of local recurrence (5.9% at 2 years) [16]. In contrast, systemic therapies such as everolimus, sunitinib, somatostatin analogs, and PRRT have convincing phase 3 data in the palliative setting [11].

In view of these increasingly robust data on the potential treatment of OMD in NET and other solid cancers, we wanted to investigate in this analysis whether OMD in NET was associated with superior survival and whether aggressive locoregional treatment of OMD portended a better prognosis. We hypothesized that insights into the frequency and course of OMD in NET could help identify patients who might benefit from such curative-intent therapies. Alternatively, as a null hypothesis, there may be no difference in prognosis between oligometastatic and polymetastatic patients, for instance, due to the generally low growth rate and perhaps early widespread seeding of tumor cells, so that systemic therapies may be preferable for most patients.

## Methods

### Patients

The study population was retrospectively selected based on patients treated at the Medical University of Vienna between January 2010 and October 2023. Patients were included in this analysis if they were diagnosed with a NET G1 or G2 of gastroenteropancreatic (GEP) origin or with a NET of unknown primary (CUP) that had likely spread from a GEP site. All patients were required to have distant metastatic disease at any point in the clinical course, with patients excluded in case of insufficient information on metastases. Parameters of interest regarding patient demographics, tumor characteristics, treatment information, and outcome were abstracted from electronic medical records, entered into a FileMaker database (Claris International Inc., Santa Clara, California, United States), and pseudonymized prior to statistical analysis. This analysis was approved by the Ethics Committee of the Medical University of Vienna (EK No: 1966/2023).

### Objectives

The main aims of this analysis were to thoroughly characterize the OMD state in NET and to assess the prognostic value of OMD in NET. The spatial and temporal distribution as well as the size and number of oligometastases were descriptively analyzed. A potential difference in OS from first metastasis and from first-line treatment initiation between oligometastatic and polymetastatic patients was evaluated.

### Definitions

OMD was defined based on the number of metastatic lesions detected on routine imaging and was limited to five metastases in up to three different organs, including distant but not local lymph nodes. Diffuse serosal spread or diffuse involvement of the bone marrow was not allowed in the OMD definition and was deemed as polymetastatic disease (PMD). Patients with oligometastases on imaging within 30 days after initial diagnosis (date of the histology report) were considered to have synchronous OMD, whereas patients who were initially nonmetastatic and were diagnosed with oligometastases after 30 days were said to have metachronous OMD. If the first staging was conducted only after 30 days and revealed OMD, this was also considered synchronous OMD (four cases).

Data on size, number, and location of metastases were extracted from radiology reports of routinely performed positron emission tomography (PET) / computed tomography (CT) / magnetic resonance imaging (MRI). These reports were written by different radiologists. If the report read “multiple metastases” and no specific number of metastases was provided, original images were reviewed to confirm the presence of more than five metastases (PMD). If this was not possible or unclear, the patient was excluded.

OS was calculated from the date of diagnosis, from the date of first metastasis, and from the start date of the first palliative systemic treatment to the date of all-cause death or to the last follow-up date in case of censoring. Progression-free survival (PFS) was calculated from the start of the first palliative systemic therapy to disease progression or death.

### Statistical analysis

All analyses were performed with R version 4.5.1 (R Foundation for Statistical Computing, Vienna, Austria) [17] and with the packages tidyverse [18], ggsurvfit [19], gtsummary [20], and ggbeeswarm [21]. Patient characteristics were summarized with medians and ranges in case of continuous variables and with frequencies and percentages in case of categorical variables. Crude statistical comparisons between oligometastatic and polymetastatic patients were performed using Fisher’s exact test and Wilcoxon rank sum test. Survival analysis according to the Kaplan-Meier method was done for OS and PFS, and subgroups were compared using the log-rank test. A two-sided *p* value < 0.05 was defined as statistically significant, with no strategy to adjust for multiple testing implemented. The sample size was not predefined but resulted from the number of patients eligible for this analysis.

## Results

### Patient characteristics

A total of 190 patients with metastatic NET of gastroenteropancreatic (GEP) or unknown (CUP) origin were included in this analysis. The study population comprised 89 (47%) female and 101 (53%) male patients. At diagnosis, the median age was 60 years, 91% were rated ECOG 0, and 80% of patients presented with stage IV, see Table 1. The majority of patients had a NET located in the small intestine (*n* = 99, 52%) or in the pancreas (*n* = 62, 33%), with further primary tumor sites being the duodenum (*n* = 6, 3.2%), the stomach (*n* = 4, 2.1%), the colon (*n* = 4, 2.1%), the rectum (*n* = 2, 1.1%), or unknown (CUP, *n* = 13, 6.8%). About one third of tumors was categorized as NET grade 1 (G1, *n* = 58, 31%) and 61% (*n* = 116) were NET grade 2 (G2), whereas *n* = 16 (8.4%) were NET G1/G2 unclassified. The median Ki-67 index was 5% (range: 0.5–20.0%). Overall, 39% exhibited functional tumor symptoms and 68% received surgery for their NET. Somatostatin analogs (SSA) were the most frequent first-line treatment (*n* = 137, 77%), followed by peptide receptor radionuclide therapy (PRRT) (*n* = 16, 9.0%), everolimus (*n* = 11, 6.2%), and other systemic therapies (*n* = 13, 7.3%), including clinical trials (*n* = 4), interferon + SSA (*n* = 3), cisplatin-etoposide (*n* = 2), capecitabine-temozolomide, sunitinib, PRRT + capecitabine, and unknown therapy (*n* = 1 each). Thirteen patients had no systemic treatment.

**Table 1:** Patient characteristics.

Parameter	*N*	Overall *N* = 190^a^	Oligometastatic *N* = 55^a^	Polymetastatic *N* = 135^a^	*p* value^b^
**Sex**	190				0.6
Female		89 (47%)	24 (44%)	65 (48%)	
Male		101 (53%)	31 (56%)	70 (52%)	
**Age at diagnosis**	190	60 (27, 82)	61 (31, 81)	60 (27, 82)	0.8
**ECOG performance score**	161				**0.011**
0		146 (91%)	48 (100%)	98 (87%)	
1		14 (8.7%)	0 (0%)	14 (12%)	
3		1 (0.6%)	0 (0%)	1 (0.9%)	
**Disease stage**	181				**<0.001**
Stage I		4 (2.2%)	2 (3.6%)	2 (1.6%)	
Stage II		9 (5.0%)	6 (11%)	3 (2.4%)	
Stage III		23 (13%)	15 (27%)	8 (6.3%)	
Stage IV		145 (80%)	32 (58%)	113 (90%)	
**Localization**	190				>0.9
Small intestine		99 (52%)	29 (53%)	70 (52%)	
Pancreas		62 (33%)	18 (33%)	44 (33%)	
CUP		13 (6.8%)	3 (5.5%)	10 (7.4%)	
Duodenum		6 (3.2%)	2 (3.6%)	4 (3.0%)	
Stomach		4 (2.1%)	1 (1.8%)	3 (2.2%)	
Colon		4 (2.1%)	2 (3.6%)	2 (1.5%)	
Rectum		2 (1.1%)	0 (0%)	2 (1.5%)	
**Grading**	190				0.3
NET G1		58 (31%)	19 (35%)	39 (29%)	
NET G2		116 (61%)	34 (62%)	82 (61%)	
NET unclassified (G1/G2)		16 (8.4%)	2 (3.6%)	14 (10%)	
**Ki-67 index (%)**	165	5.0 (0.5, 20.0)	4.0 (0.5, 19.0)	5.0 (1.0, 20.0)	0.6
**Functionality**	179	70 (39%)	16 (31%)	54 (42%)	0.2
**Surgery**	190	130 (68%)	46 (84%)	84 (62%)	**0.004**
**First-line treatment**	177				0.5
Everolimus		11 (6.2%)	3 (5.9%)	8 (6.3%)	
Other		13 (7.3%)	6 (12%)	7 (5.6%)	
PRRT		16 (9.0%)	3 (5.9%)	13 (10%)	
SSA		137 (77%)	39 (76%)	98 (78%)	

^a^n (%); Median (Min, Max).

^b^Fisher’s exact test; Wilcoxon rank sum test.

Statistically significant values are marked in bold.

At initial diagnosis, 145/186 (78%) of patients presented with distant metastases, mainly to the liver (73%), but also to distant lymph nodes (19%), bones (11%), peritoneum (7.0%), lung (2.2%), and other sites (4.3%), see Table 2. Local lymph node metastasis were present in 74% of patients. At any point in the follow-up, all patients had distant metastasis. Again, most frequently, the liver was affected by metastases (92%), followed by distant lymph nodes (45%), bone (39%), peritoneum (19%), lung (15%), and other sites (14%). Metastases to local lymph nodes occurred in 10 more patients (*n* = 137/190, 72%). No brain metastases were documented.

**Table 2:** Metastatic sites in oligometastatic versus polymetastatic disease

Metastatic site	*N*	Overall *N* = 190^a^	Oligometastatic *N* = 55^a^	Polymetastatic *N* = 135^a^	*p* value^b^
**Distant metastasis at diagnosis**	186	145 (78%)	32 (58%)	113 (86%)	**<0.001**
Liver	184	134 (73%)	31 (57%)	103 (79%)	**0.004**
Lung	183	4 (2.2%)	1 (1.8%)	3 (2.3%)	>0.9
Bone	185	21 (11%)	1 (1.8%)	20 (15%)	**0.005**
Brain	181	0	0	0	
Peritoneal carcinomatosis	185	13 (7.0%)	0 (0%)	13 (10%)	**0.011**
Other site	186	8 (4.3%)	1 (1.8%)	7 (5.3%)	0.4
Distant lymph nodes	178	34 (19%)	6 (11%)	28 (22%)	0.10
**Local lymph nodes at diagnosis**	172	127 (74%)	34 (65%)	93 (78%)	0.13
**Distant metastasis at any time point**	190	190 (100%)	55 (100%)	135 (100%)	
Liver	190	175 (92%)	50 (91%)	125 (93%)	0.8
Lung	190	29 (15%)	9 (16%)	20 (15%)	0.8
Bone	190	75 (39%)	20 (36%)	55 (41%)	0.6
Brain	190	0	0	0	
Peritoneal carcinomatosis	190	37 (19%)	8 (15%)	29 (21%)	0.3
Other site	190	27 (14%)	7 (13%)	20 (15%)	0.8
Distant lymph nodes	190	85 (45%)	22 (40%)	63 (47%)	0.4
**Local lymph nodes at any time point**	190	137 (72%)	40 (73%)	97 (72%)	>0.9

^a^n (%).

^b^Fisher’s exact test.

Statistically significant values are marked in bold.

### Comparison of oligometastatic and polymetastatic patients

Among the 190 patients, 55 (29%) patients were classified as oligometastatic at any time point during the clinical course. Comparisons of general patient characteristics regarding this metastatic state showed that oligometastatic patients had a significantly higher ECOG status (ECOG 0 in 100% versus 87% in polymetastatic patients, *p* = 0.011) and a significantly lower disease stage at initial diagnosis (stage IV in 58% versus 90%, *p* < 0.001). Furthermore, a higher fraction of oligometastatic patients received surgery of the primary tumor (84% versus 62%, *p *= 0.004), see Table 1. There were no significant differences regarding sex, age, localization, grading, Ki-67 index, functionality, and first-line treatment between oligometastatic and polymetastatic patients.

### Characterization of the oligometastatic state

**Fig. 1:**
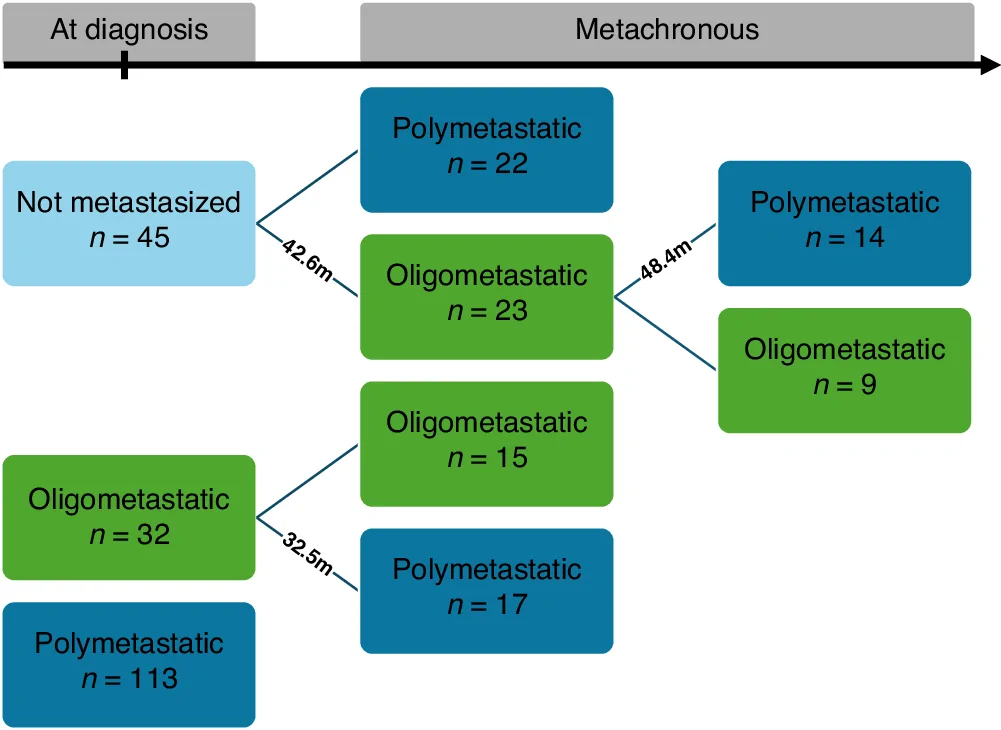
Metastatic state at diagnosis and throughout the course of the disease. Not metastasized (*n* = 45) includes 4 patients with missing information on setting at diagnosis. Time (in months) until change of disease state is given as median.

Of the 55 patients with oligometastatic disease, 32/55 (58%) had synchronous oligometastases, whereas 23/45 (51%) patients who were nonmetastatic at diagnosis developed oligometastases metachronously after a median of 42.6 months. Of the overall cohort, 17% (*n* = 32/190) were oligometastatic at diagnosis. At the end of the observation period, 24/55 (44%) patients with oligometastases remained oligometastatic. In comparison, 31 patients developed further metastases that terminated the OMD state after a median of 32.5 months (oligometastatic at diagnosis) or 48.4 months (nonmetastatic at diagnosis), see Fig. 1. No significant differences in the clinical characteristics from Table 1 were observed between OMD patients who remained at this stage and those who transitioned to the PMD stage, except in sex (75% versus 43% male, *p* = 0.027), disease stage (13% versus 39% stage 3, *p* = 0.044), and first-line treatment (SSA in 100% versus 61%, *p* = 0.008).

**Fig. 2:**
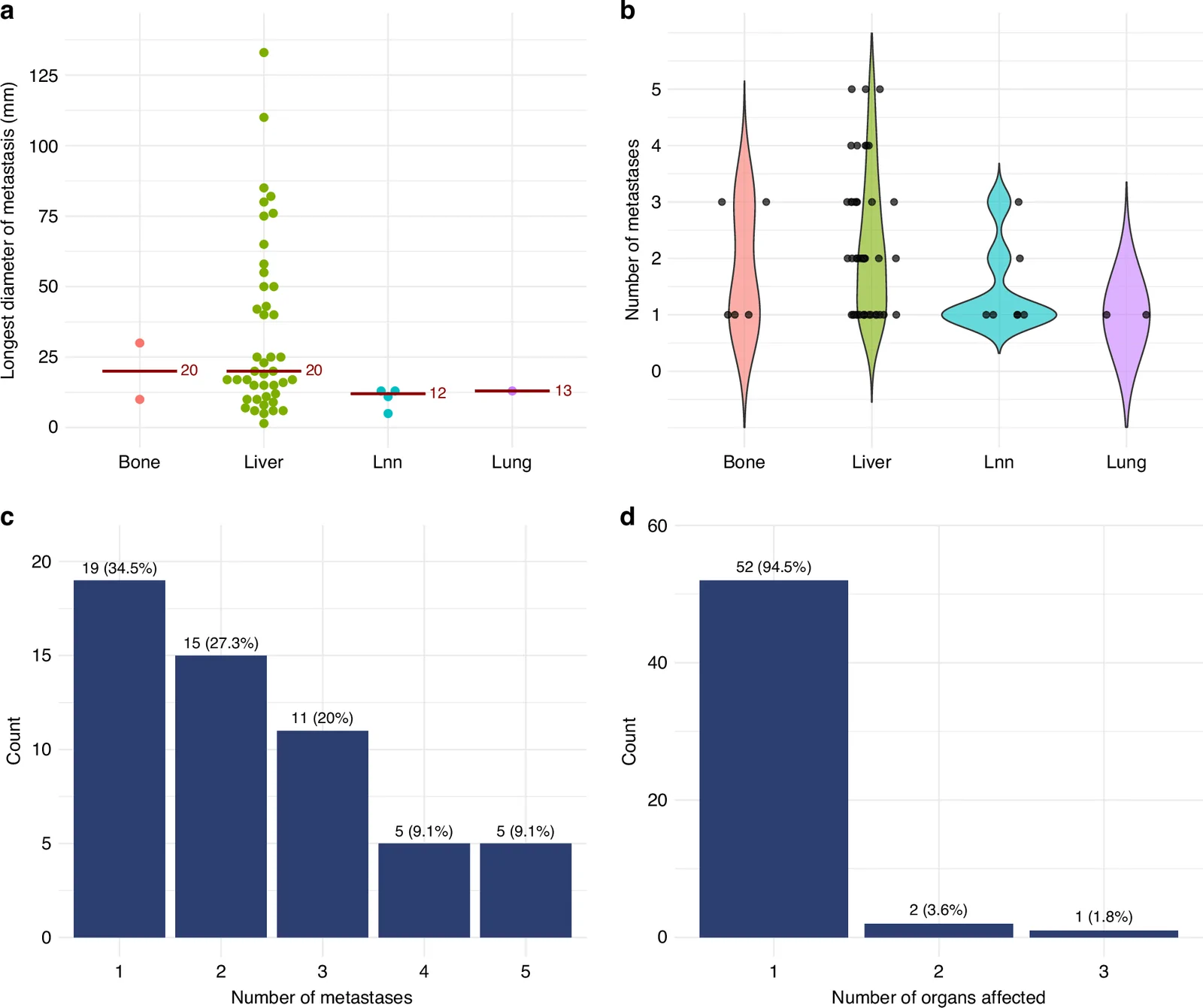
Characteristics of oligometastases. Longest diameter of metastasis in mm according to affected organ, with the medians marked by a red line (**a**). Number of metastases according to metastatic site (**b**). Bar plot showing how many patients had a certain number of metastases (**c**). Bar plot of the number of organs affected in oligometastatic patients (**d**).

Oligometastases were most commonly present in the liver. The median longest diameter of metastases to the liver was 20 mm (range: 1.5–133 mm, *n* = 43, *n* = 12/43 or 28% with ≥50 mm), for lymph node metastases 12 mm (range: 5–13 mm, *n* = 4), for bone metastases 20 mm (range: 10–30 mm, *n* = 2), and 13 mm for lung metastases (*n* = 1), see Fig. 2a. Patients oligometastatic to the liver showed a higher number of metastases (8/46 or 17% had 4–5 hepatic metastases) than patients with oligometastases in the bones, lymph nodes, or lungs (all ≤3), see Fig. 2b. However, about one third of oligometastatic patients had only one metastasis (monometastatic), whereas 27% had two, 20% had three, and 9.1% each had 4 or 5 metastases, see Fig. 2c. Most oligometastatic patients (95%) had only one organ that was affected by metastases (mostly the liver in *n* = 43/52 or 83%), with only two patients and one patient having oligometastases in two or three organs, respectively, see Fig. 2d.

### Survival analyses

For the total patient cohort, the median OS from diagnosis was 117.5 months (95% CI 102.4–149.1 months), from occurrence of the first metastasis 102.4 months (95% CI 98.3–117.5 months), and from treatment start 90.2 months (95% CI 76.9–113.2 months). The median PFS from first-line palliative treatment was 26.8 months (95% CI 18.4–39.0 months). The median follow-up after diagnosis was 114.2 months (95% CI 86.4-134.5 months) according to reverse Kaplan-Meier.

**Fig. 3:**
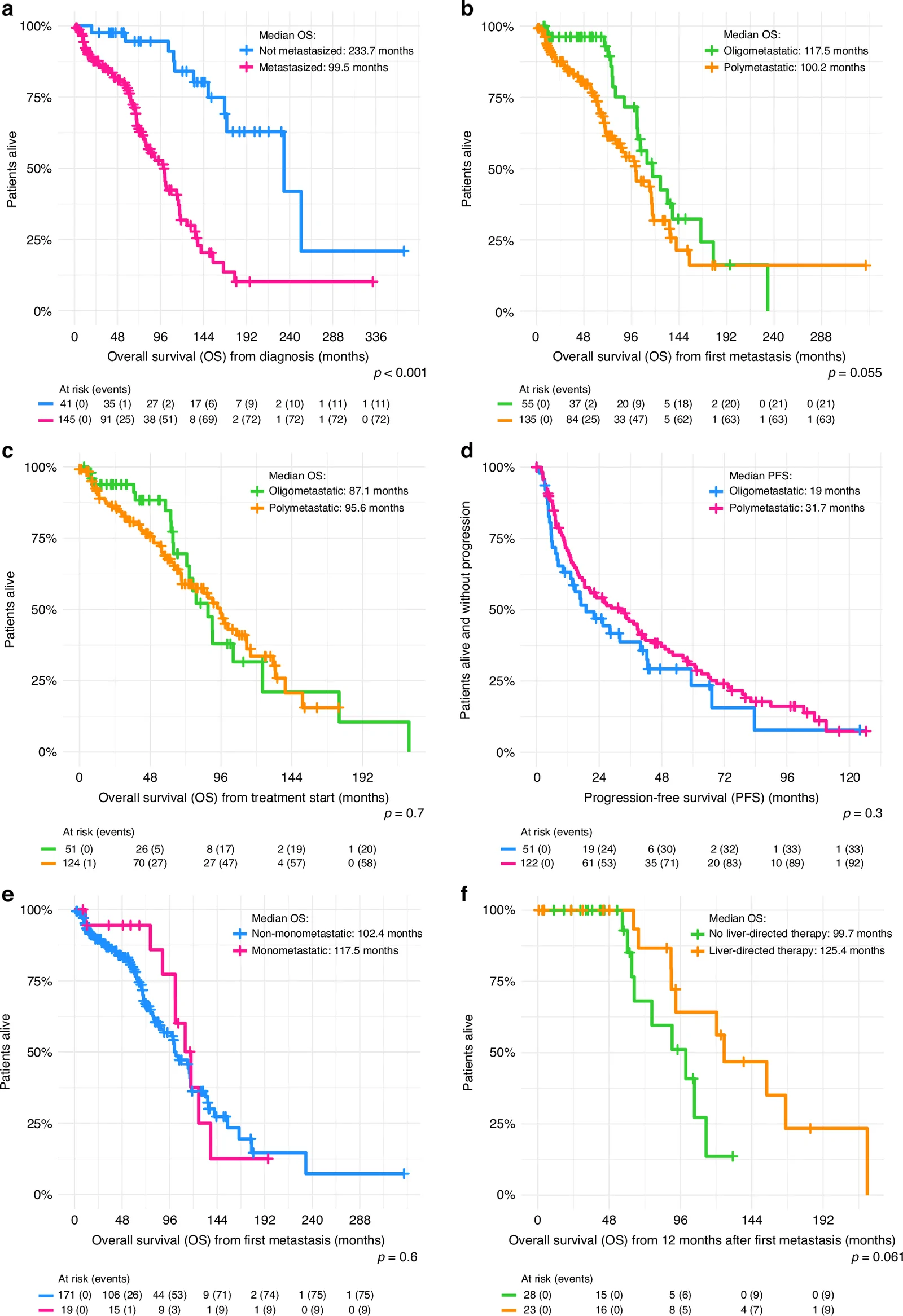
Survival analyses. Kaplan-Meier analysis of OS from diagnosis comparing patients with and without metastasis at diagnosis (**a**). Comparisons between oligometastatic and polymetastatic patients in terms of OS from first metastasis (**b**), from treatment start (**c**), and of PFS from first-line treatment start (**d**). Kaplan-Meier analysis comparing OS from first metastasis between monometastatic and non-monometastatic patients (**e**). Comparison between oligometastatic patients with versus without liver-directed therapy (**f**) regarding OS from landmark time point.

There was a significant difference (*p* < 0.001) in OS from diagnosis between patients who were metastatic at diagnosis (median OS of 99.5 months, 95% CI 79.7–116.0 months) and patients who initially had no distant metastases (median OS of 233.7 months, 95% CI 169.8–not calculable [NC] months), see Fig. 3a. Likewise, there was also a significant difference (*p* < 0.001) in prognosis according to stage at diagnosis (median OS of 252.7 months for stage I, not available for stage II, 169.8 months for stage III, and 99.5 months for stage IV). Moreover, patients with ECOG 0 at diagnosis had a significantly (p < 0.001) longer OS from diagnosis (median OS of 125.3 months, 95% CI 110.6–154.5 months) than patients with ECOG ≥ 1 (median OS of 55.1 months, 95% CI 10.0–NC months). Patients who had primary tumor resection showed a significantly (*p* < 0.001) superior OS (median OS of 134.4 months, 95% CI 116.0–252.7 months) than patients without surgery (median OS of 72.4 months, 95% CI 62.0–NC months).

Patients with oligometastatic NET (synchronous or metachronous, *n* = 55) had a numerically longer median OS from first metastasis (117.5 months, 95% CI 101.8–NC months) than patients with de novo polymetastatic NET (100.2 months, 95% CI 80.5–116.9 months), with this difference not reaching statistical significance (*p* = 0.055), see Fig. 3b. No difference was found between oligometastatic patients (median OS of 87.1 months, 95% CI 72.7–NC months) and polymetastatic patients (median OS of 95.6 months, 95% CI 69.4–116.1 months) in terms of OS from treatment start, see Fig. 3c. Furthermore, oligometastatic patients reached a median PFS of 19 months (95% CI 13.1–42.6 months), which was numerically but not significantly (*p* = 0.3) shorter than the median PFS achieved by polymetastatic patients (31.7 months, 95% CI 18.4–41.7 months), see Fig. 3d. Having only a single metastasis was not associated with significantly better survival, with monometastatic patients achieving a median OS from first metastasis of 117.5 months (95% CI 101.7–NC months) and non-monometastatic patients attaining a median OS of 102.4 months (95% CI 87.1–117.1 months), see Fig. 3e.

### Locoregional treatment of oligometastases

Of the 55 oligometastatic patients, 24 had locoregional therapies. Twenty-two had surgical resection of liver metastases after a median of 2.1 months from first metastasis (range: 0–35.1 months), and 6 patients had other liver metastasis-directed treatments, including selective internal radiotherapy (SIRT, *n* = 3), embolization (*n* = 2), radiofrequency ablation (RFA, *n* = 1), and transarterial chemoembolization (TACE, *n* = 1). Over the entire follow-up period, 11/24 patients (46%) with locoregional treatment versus 20/31 patients (65%) without metastasis-directed therapy had transitioned to PMD (*p* = 0.184). At the landmark time of 12 months after first metastasis, 23 patients had received liver-directed therapy (*n* = 21 with surgery and *n *= 3 with other locoregional therapy), whereas 28 had not, and four patients had been excluded. There was a numerical but no significant difference in OS between oligometastatic patients who had received liver-directed therapies within 12 months (median OS of 125.4 months, 95% CI 92.9–NC months) and patients who had not received such treatments for oligometastases in the liver (median OS of 99.7 months, 95% CI 65.0–NC months), see Fig. 3f.

## Discussion

This analysis compared patients with up to five metastasis (oligometastatic) and patients with more than five metastases (polymetastatic) in terms of patient characteristics and prognosis. Whereas the presence of metastases at diagnosis was associated with a significantly reduced OS (median OS of 99.5 months versus 233.7 months), there was only a trend (*p* = 0.055) toward a difference in OS from first metastasis between patients with few versus many metastases (median OS of 117.5 months versus 100.2 months). After a median follow-up of 114.2 months, more than half of patients with OMD (56%) had transitioned into a PMD state. Notably, oligometastatic patients more frequently had baseline characteristics that were associated with longer survival, i.e., better ECOG status, lower stage disease, and primary tumor resection. In the subgroup of oligometastatic patients, liver-directed therapy within 12 months after first metastasis was linked to a numerically longer OS compared to no such treatment (125.4 months versus 99.7 months), with this difference not reaching statistical significance. However, this indicates that a selected subgroup of OMD patients might derive a benefit from locoregional treatment.

We characterized the OMD state in NET and found that 17% of included patients or 22% of initially metastatic patients were oligometastatic at diagnosis. This aligns with data from a population-based registry study using initial Ga68-DOTATATE staging scans, which reported OMD in 9.2% of all analyzed patients or 20% (*n* = 30/151) of metastatic patients [22]. The metastatic pattern of NET are different depending on primary tumor site [23] and it is noteworthy that most of our GEP-NET patients (almost 95%) with OMD had metastases in only one organ, which was the liver in 83% of cases. In contrast, bone-only or lung-only OMD in NET is rare. This highlights the value of liver-directed therapies within a potential OMD treatment concept in NET. Moreover, the diameter of the largest metastases in an oligometastatic patient can be rather large in some patients (e.g., *n* = 12/43 with a liver metastasis sized 50–130 mm), which can be relevant for the choice of local therapy.

The conceptual model of OMD with the notion of offering radical and potentially curative treatment to patients with metastases is intriguing. As far as data are currently available, most randomized controlled trials of patients with varying histologies suggested a benefit from ablative treatment [24]. Accordingly, it remains a clinically relevant question whether patients with NET presenting with OMD should also be considered for dedicated therapeutic strategies. Addressing this issue is particularly challenging given the considerable biological and clinical heterogeneity within this patient population. Based on the similar prognosis of oligometastatic and polymetastatic patients in our analysis, a reasonable interpretation could be that the absolute number of metastases plays a minor role, possibly because NET metastases tend to grow rather slowly [25] and because most oligometastases might only qualify for palliative-intent treatment. As a consequence, systemic therapies would be preferable for most patients irrespective of metastasis count. In support of this, there was also no survival advantage in patients with a single metastasis (*n* = 19/55 or 35% of patients with OMD). Alternatively, one could argue that OMD is only prognostically favorable if appropriate locoregional treatment is initiated, which was attempted in only 45% (*n* = 23/51) of patients within 12 months after first metastasis but might have been feasible in more patients. Implementing such an approach necessitates, first, that all patients undergo rigorous and standardized staging procedures and, second, that within the OMD cohort, those individuals with particularly favorable prognostic profiles are reliably identified. Hence, the number of metastases might be less important than the (curative) treatability of these metastases, as there might be, for instance, patients with many more than five liver metastases but with the chance of complete removal of all lesions. To provide conclusive evidence on this, an OMD-specific treatment protocol needs to be evaluated in a randomized controlled study. For instance, OligoRARE (NCT04498767) is a phase 3 trial comparing standard of care alone versus standard of care in combination with SABR in patients with rare oligometastatic cancers (i.e., excluding breast, prostate, lung, or colorectal cancer) [26].

Survival benefits may potentially be derived from liver-directed therapy. In a 2022 systematic review of 32 studies on the treatment of neuroendocrine liver metastases, the outcome of surgery (*n* = 2521 patients, 5-year OS rate of 67%, 5-year disease-free survival [DFS] rate of 30.2%) compared favorably with liver transplantation (*n* = 705, 5-year OS rate of 60%, 5-year DFS rate of 20%) and ablation/embolization techniques (*n* = 916, 5-year OS rate of 36.2%, 5-year DFS rate 19%) [27]. The extent to which patient selection plays a role is unknown, since none of these trials were randomized [27], and eligibility criteria for the respective treatments may vary considerably. In one notable analysis of a prospective database (*n* = 649 patients with liver metastases), the longest median OS was observed with hepatic resection (160 months) and radiofrequency ablation (123 months), which were both performed in highly selected cohorts (*n* = 58 and *n* = 28 or 9% and 4%, respectively), as compared to the larger subgroups of systemic therapy (*n* = 316, 70 months), chemoembolization (*n* = 130, 66 months), and observation (*n* = 117, 38 months) [28]. Recently, initial results of a randomized trial called RETNET (*n* = 151), a comparison of transarterial bland embolization (TAE), conventional transarterial chemoembolization (cTACE), and drug-eluting bead transarterial chemoembolization (DEB-TACE), were published, showing high toxicity with DEB-TACE and no significant difference in hepatic PFS between cTACE (13 months) and TAE (18 months) [29]. In our analysis, evaluation of the prognosis associated with liver-directed therapy was restricted to the group of oligometastatic patients. Compared to patients without locoregional treatments, the OS difference was borderline non-significant, likely influenced by the small sample size, cohort heterogeneity, and the retrospective study design. With respect to the OMD hypothesis, these results are inconclusive.

Novel blood-based biomarkers such as circulating tumor cells (CTC) or circulating tumor DNA (ctDNA) might aid in the diagnosis of OMD. Liquid biopsy could be useful in cases of apparent OMD but radiographically undetectably micrometastases, as this approach might enable identification of patients who are not truly oligometastatic and who thus would not be disease-free after definitive treatment of all evident lesions. One conclusion from the SABR-COMET study was that most oligometastatic patients have occult micrometastases, a finding that was based on comparable times to new metastases across the two study arms, the frequent need for salvage SABR, and a relatively short PFS benefit [10]. In a 2010 pathology study comparing meticulous histologic assessment of 11 hemi-hepatectomy specimens (3–4-mm thick serial slices) with preoperative imaging found that less than half of the NET metastases had been detected preoperatively (mean accuracy of 49% for MRI, 38% for CT, and 24% for somatostatin receptor scintigraphy) [30]. With regard to liquid biopsy and NET tumor load, there are currently only very limited data available [31, 32]. From prior studies of locoregional therapies it is clear that patient selection is key, and liquid biopsy may improve this process. At present, Ga68-DOTATATE PET is a very sensitive method to detect small metastases early [33] and could be considered the method of choice to determine OMD status. Even if this approach may potentially detect small, clinically irrelevant or asymptomatic metastases, the use of the most sensitive imaging modality appears justified, particularly prior to exclusively local therapy.

Several limitations of this analysis have to be acknowledged. This was a retrospective review of electronic health records from patients with metastatic NET treated at a tertiary referral center. Hence, OMD assessment was based on radiology reports from routine imaging studies. These imaging exams were performed in accordance with relevant guidelines but inherently lacked standardized reporting and evaluation of potential OMD and suffer from heterogeneity in terms of multiple observers and imaging modalities, with no review by an expert radiologist as part of this study. Furthermore, our real-world experience lacked a specific OMD treatment protocol and is also limited by the relatively small sample size (*n* = 190) and multiple testing without an adjustment procedure.

To conclude, our data demonstrated non-significant trends for a better outcome in oligometastatic NET patients and in patients receiving liver-directed therapy. Given the slow growth rate and the likelihood of micrometastases, it seems questionable whether a disease-free state is achievable with liver-directed therapies in a relevant fraction of NET patients with OMD or whether tumor volume reduction (in nonfunctional NET) has a significant role in view of an increasing number of systemic treatment options. Further evidence, optimally from a randomized trial of an OMD treatment strategy, is necessary.

## Data Availability

Further data and the computer code for statistical analysis may be available at reasonable request to the corresponding author.
